# Quantifying Integrated Proteomic Responses to Iron Stress in the Globally Important Marine Diazotroph *Trichodesmium*

**DOI:** 10.1371/journal.pone.0142626

**Published:** 2015-11-12

**Authors:** Joseph T. Snow, Despo Polyviou, Paul Skipp, Nathan A. M. Chrismas, Andrew Hitchcock, Richard Geider, C. Mark Moore, Thomas S. Bibby

**Affiliations:** 1 Ocean and Earth Science, National Oceanography Centre Southampton, University of Southampton, Southampton, United Kingdom; 2 Stem Cell and Leukaemia Proteomics Laboratory, Manchester Academic Health Science Centre, The University of Manchester, Wolfson Molecular Imaging Centre, Manchester, United Kingdom; 3 Centre for Proteomic Research, University of Southampton, Southampton, United Kingdom; 4 School of Geographical Sciences, University of Bristol, University Road, Clifton, Bristol, United Kingdom; 5 Department of Molecular Biology and Biotechnology, University of Sheffield, Firth Court, Western Bank, Sheffield, United Kingdom; 6 School of Biological Sciences, University of Essex, Colchester, United Kingdom; Mount Allison University, CANADA

## Abstract

*Trichodesmium* is a biogeochemically important marine cyanobacterium, responsible for a significant proportion of the annual ‘new’ nitrogen introduced into the global ocean. These non-heterocystous filamentous diazotrophs employ a potentially unique strategy of near-concurrent nitrogen fixation and oxygenic photosynthesis, potentially burdening *Trichodesmium* with a particularly high iron requirement due to the iron-binding proteins involved in these processes. Iron availability may therefore have a significant influence on the biogeography of *Trichodesmium*. Previous investigations of molecular responses to iron stress in this keystone marine microbe have largely been targeted. Here a holistic approach was taken using a label-free quantitative proteomics technique (MS^E^) to reveal a sophisticated multi-faceted proteomic response of *Trichodesmium erythraeum* IMS101 to iron stress. Increased abundances of proteins known to be involved in acclimation to iron stress and proteins known or predicted to be involved in iron uptake were observed, alongside decreases in the abundances of iron-binding proteins involved in photosynthesis and nitrogen fixation. Preferential loss of proteins with a high iron content contributed to overall reductions of 55–60% in estimated proteomic iron requirements. Changes in the abundances of iron-binding proteins also suggested the potential importance of alternate photosynthetic pathways as *Trichodesmium* reallocates the limiting resource under iron stress. *Trichodesmium* therefore displays a significant and integrated proteomic response to iron availability that likely contributes to the ecological success of this species in the ocean.

## Introduction

The low (bio-)availability of iron (Fe) in oxic seawater [1] appears to play a key role in controlling the distribution and activity of oceanic diazotrophic cyanobacteria [2–6]. The metabolic pathways of N_2_-fixation and oxygenic photosynthesis both have a high absolute requirement for Fe, together accounting for the majority of Fe in diazotrophic cyanobacteria [7,8]. *Trichodesmium spp*. likely represent the most abundant marine diazotrophic cyanobacteria, with the widespread distribution of this genus throughout the surface tropical oceans estimated to supply 60–80 of the 100–200 Tg N yr^-1^ of annual global oceanic N_2_ fixation [9,10]. However, the availability of Fe appears to be a key control on the broad-scale biogeography and activity of *Trichodesmium spp*. [11] and, potentially, the overall contribution of this genus to global N_2_-fixation.

N_2_-fixation and oxygenic photosynthesis can be considered antagonistic due to the potential for irreversible inactivation of the enzyme catalyzing N_2_-fixation, nitrogenase, by molecular oxygen (O_2_) produced as a photosynthetic by-product [12]. Diazotrophic cyanobacteria typically mitigate against this antagonism through the spatial segregation of N_2_-fixation into anoxygenic, non-photosynthetic cells termed heterocysts [13], or through undertaking N_2_-fixation at night [14]. However *Trichodesmium spp*. simultaneously perform N_2_-fixation and photosynthesis during the day without complete spatial or temporal segregation [15–18]. Partial and reversible differentiation of a subset of cells along the filament into ‘diazocytes’ with elevated localized N_2_-fixation capacity [16,19], coupled with finely regulated control of photosynthesis and N_2_-fixation within the photoperiod [16] appears to facilitate this daytime N_2_ fixation, but likely places an increased Fe requirement on *Trichodesmium spp*. [16,20].

*Trichodesmium spp*. can readily adapt their proteome [7,21,22], transcriptome [23] and elemental composition [24,25] in response to environmental forcings. To determine the molecular-level response of *Trichodesmium* to Fe stress, targeted studies have identified several key molecular adaptations. These include: expression and accumulation of the chlorophyll-binding Fe-stress-induced protein IsiA [7,26]; the Fe-deficiency-induced protein IdiA [27]; the soluble electron carrier IsiB (flavodoxin) [28], which functionally replaces Fe-binding ferredoxin [29]; a reworking of the photosynthetic and nitrogenase apparatus [7,26]; and changes in Fe uptake and storage processes [29]. Whilst these previous studies have identified specific genes/proteins that change in abundance in relation to Fe stress, no holistic analysis of the coordinated proteomic response to Fe stress has been performed to date.

Here we present label-free quantitative proteomic profiles from a controlled Fe stress study on *T*. *erythraeum* IMS101 cultures. Using ultra-performance liquid chromatography-mass spectrometry (1D-UPLC-MS^E^) [30,31] we describe and quantify whole-proteome-scale coordinated responses to Fe stress in *Trichodesmium*, with a specific focus on the Fe-binding proteins.

## Materials and Methods

### Culture Conditions

*Trichodesmium erythraeum* strain IMS101 was grown in filter sterilized, modified YBC-II growth media [32]. Trace metal and EDTA concentrations were modified to produce a well-defined trace metal chemistry in line with those of [33,34]. Specifically, EDTA concentrations were increased to 20 μM with amended final concentrations of Cu—8 nM, Zn—20 nM, Co—8 nM, Mn—18 nM, Mo—100 nM, Ni—20 nM, Se—10 nM. Total added Fe concentrations were 0 nM, 120 nM and 400 nM, the latter two conditions corresponding to estimated inorganic Fe concentrations of ~350 and 1100 pM Fe’ respectively [34,35]. Batch cultures were grown in 25 cm^3^ 0.2 μm vented sterile polystyrene flasks (Corning Inc., NY, USA). Growth conditions were maintained at 27°C, 12:12 hour light:dark cycle at 100–160 μmol photons m^-2^ s^-1^ and subject to gentle agitation using an orbital shaker.

Inoculations of experimental cultures were performed from late exponential phase cells grown at 160 nM total added Fe. Inoculant was pre-concentrated onto 5 μm track etched polycarbonate membranes (Nucelopore, Whatman, Kent, UK), washed with Fe-free modified YBC-II growth media. Cells were then re-suspended in a small volume of Fe-free growth media before being used to inoculate experimental flasks. Fe-replete (Fe+, 400 nM added Fe), Fe-deplete (Fe-, 0 nM added Fe) and Fe re-fed (initially 0 nM, later 400 nM) cultures were then established. Sets of triplicate cultures from each treatment were used for daily physiological assessment to establish Fe stress whilst additional biological triplicate cultures for proteomic analysis remained sealed throughout the experiment to minimize potential Fe contamination.

### Photosynthetic Physiology

Daily assessment of photosynthetic physiology was performed 2.5 hours after the onset of the photo-period using a FASTtracka MkII Fast Repetition Rate fluorometer (FRRf) integrated with a FastAct™ Laboratory system (Chelsea Technologies Group Ltd, Surrey, UK). Cells were dark acclimated for 20 minutes upon sampling and then maintained for 5 minutes at a low constant irradiance of 21 μmol photons m^-2^ s^-1^ immediately prior to measurement to reverse the influence of any reduction of the inter-photosystem electron transport chain in the dark [7]. The photophysiological parameter F_v_/F_m_ was used as an estimate of the apparent photosystem II (PSII) photochemical quantum efficiency [36]. Analyses were performed in triplicate with absolute values of F_v_/F_m_ being gain and blank corrected using cell-free growth media.

### Cell counts

Cell counts were performed using a Sedgewick rafter counting chamber. Photographs were taken using a GX CAM-1.3 camera (GT Vision Ltd, Suffolk, UK) on an L1000A biological microscope (GT Vision Ltd, Suffolk, UK). Cell and filament lengths were quantified using GX capture software (GX Optical, Suffolk, UK) and the number of cells per filament was calculated by dividing the total filament length by the average cell length, with cells per ml then calculated as the product of total filament counts and cells per filament. Cell counts were performed in triplicate for each treatment every 48 hours. Cell counts were subsequently used to derive growth rates under the different conditions.

### Statistical Analysis of Culture Data

Data collected for cell counts and F_v_/F_m_ were subject to outlier identification and removal following Grubbs' test for outliers. A Student's t-test was used to determine statistical significance (n = 3, P<0.05).

### Protein Preparation and Digestion

Proteomic sampling was performed on day 7 during early exponential growth when cultures displayed maximum divergence in Fe stress physiology. Samples from Fe+ and Fe- cultures were collected 2 hours (T1+ and T1-) and 6 hours (T2+ and T2-) after the onset of the photoperiod to partially account for any alteration in Fe stress responses and cellular Fe requirements over the photoperiod [37]. Cultures were filtered onto glass-fibre filters (Whatman) before being snap-frozen in liquid nitrogen and stored at -80°C.

Protein extraction was performed in 500 μL 1x protein extraction buffer (1x PEB) [7]. Protein and filters were separated using 30 μm pore size polyethylene centrifuge filters (Pierce no. 89868, Thermo Scientific). A 200 μL aliquot of extracted protein was acetone precipitated at 4:1 acetone:sample at -20°C for 1 hour. Samples were centrifuged (10 minutes at 13,000 x g), an aliquot of the supernatant preserved for determination of chlorophyll concentration [38], and the protein pellet re-suspended in 40 μL 1x PEB. Precipitated protein was isolated using a Novex NuPAGE 10% polyacrylamide gel [39]. A gel band containing all sample protein was excised and trypsin digested [40].

Peptide concentrations following digestion were assessed using Millipore Direct Detect, suggesting an overall protein recovery from digestion of ~20–43%. Samples were subsequently biomass normalized and spiked with reference peptides (Rabbit Glycogen Phosphorylase P00489, Yeast Enolase P00924) prior to MS analysis.

### 1D-UPLC-MS^E^ Proteomics

Separations were performed using a nanoAcquity UPLC system (Waters). Prepared protein lysates (1.5 μl, 500 ng on column) containing 100 fmol of the digested Phosphorylase B and Enolase internal standards were injected onto a Symmetry C18, 180 μm x 20 mm trapping cartridge (Waters). After 5 minutes washing of the trap column, peptides were separated using a 75 μm ID x 200 mm, 1.7 μm BEH130 C18 column (Waters) using a linear gradient of 5 to 40% B (buffer A = 0.1% formic acid in water, buffer B = 0.1% formic acid in acetonitrile) over 90 minutes with a wash to 85% B at a flow rate of 300 nl/min. All separations were automated and performed on-line to the mass spectrometer.

Mass spectrometry was performed using a Waters G2-S HDMS mass spectrometer operating in MS^E^ mode with ion mobility enabled. Data was acquired from 50 to 2000 m/z using alternate low and high collision energy (CE) scans. Low CE was 5 V and elevated, ramped from 20–40 V. The lock mass Glu-fibrinopeptide, (M+2H^+2^, m/z = 785.8426) at a concentration of 100 fmol/μl was infused at 350 nl/min and acquired every 60 seconds. Technical triplicate UPLC-MS^E^ analyses were performed on each biological triplicate sample.

### Data Processing and Statistical Analysis

LC-MS^E^ data were processed using Protein Lynx Global Server (PLGS) version 2.5 for submission to the IDENTITY^E^ search engine (Waters Corporation, Milford, MA). Briefly, LC-MS^E^ spectra were lockmass corrected, centroided, deisotoped and charge state reduced and intensity measurements reduced [41]. Precursor and fragment ions for each detected compound were expressed as an accurate mass retention time (AMRT) pair.

Processed LC-MS^E^ data were submitted to the IDENTITY^E^ database search algorithm version 2.5. Details of the search strategy can be found in [41] and [42]. Each processed file was searched against a protein translation of the *T*. *erythraeum* IMS101 genome sequence acquired from http://www.uniprot.org using the following search parameters: automatic precursor and product ion tolerances were set; enzyme specificity was set to tryptic; fixed modifications included cysteine alkylation to carbamidomethyl; variable modifications included N-terminal acetylation and methionine oxidation. A maximum of 2 missed cleavages were allowed. A false discovery rate of 4% was applied. Peptide assignments were based on ≥3 fragment ions with subsequent protein assignment performed on the basis of ≥3 different identified peptides. Protein quantification was performed using the Top 3 Protein Quantification (T3PQ) method as described in [30,31]. Briefly, protein quantities in each sample were calculated as the average of the intensities of the three most abundant peptides for that given protein provided all three were observed within a threshold concentration [31]. The resulting ‘protein intensities’, representative of the protein abundance, were then converted to protein concentrations using the 100 fmol rabbit Glycogen Phosphorylase primary standard. Quantification of the secondary 100 fmol yeast Enolase standard using the same method (90 ± 6 fmol on column) indicated an accuracy within 10% and good reproducibility between analytical runs.

Individual protein concentrations were normalized between samples by dividing the observed concentration of the protein in question with the sum of all protein concentrations observed. Such normalization mitigated variability between sample replicates facilitating more robust statistical comparison. Mean concentration values for each protein were calculated from the three technical replicates, statistical significance (P<0.05) was then determined from the mean values from each of the three biological replicates analysed per experimental condition using a two-way ANOVA statistical method with a Bonferroni correction to account for multiple comparisons. Biological replicates typically demonstrated strong reproducibility with good correlations observed for all comparisons. However, one biological replicate from each of the T2- (T2- 1) and T1- (T1- 3) treatments was omitted from statistical analysis (S1 Fig). Biological replicate 'T1- 3' showed consistently reduced protein abundance when compared with T1- 1 and T1- 2. Biological replicate T2- 1 showed poor correlation to both T2- 2 and T2- 3 (R^2^ < 0.95 in both comparisons). For proteins where no data were collected for a given treatment (i.e. proteins that were absent or below detection levels in all biological replicates for a particular condition), no statistical analysis was performed and a presence/absence response was recorded. For treatments where no data was observed for 1 or 2 of the biological replicates, analysis was performed using the method outlined in [43] where ANOVA analysis is replaced with multiple regression analysis. The data-independent mode of acquisition used in this study helps mitigate the stochastic nature of data-dependent proteomics. However, certain proteins may remain unquantified as ≥3 proteotypic peptides per protein, all measured at similar concentrations, were considered necessary for confident detection. Unquantified proteins may thus be absent in the proteome, but may also not be readily detectable using the 1D-UPLC-MS^E^ method, or may have undergone post-translational modification, preventing annotation using the *T*. *erythraeum* IMS101 genome.

### Identification of Fe-containing proteins

The amino-acid sequences of the identified proteins were submitted to the PHYRE2 protein fold recognition server [44] to identify homologous Fe-binding protein structures. Sequences where protein structures matched with a confidence >95% were then searched using MetalPDB [45]. Predicted proteomic Fe concentrations were subsequently derived from the inferred Fe-stoichiometry and the observed protein concentration.

## Results and Discussion

### Physiological and proteomic sampling

**Fig 1:**
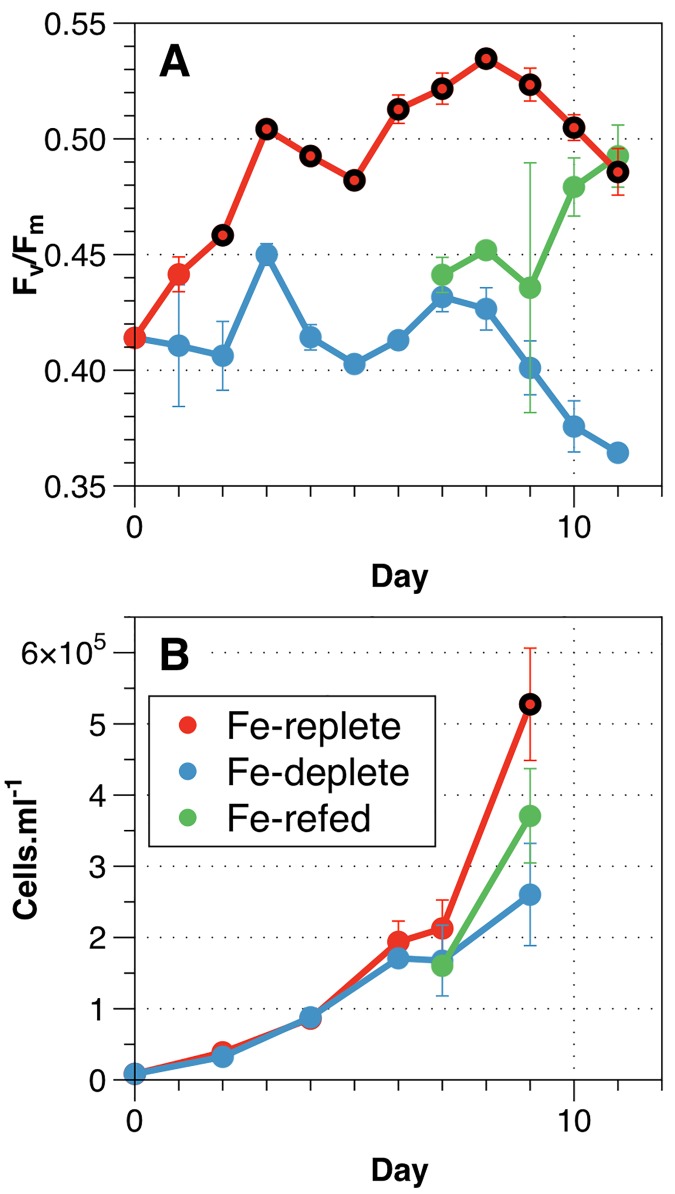
Culture physiology data. Physiological growth parameters observed throughout the culture experiment showing Fe-replete (red), Fe-deplete (blue) and Fe re-fed (green) samples. A) Photosynthetic efficiency (F_v_/F_m_). B) Cell counts (cells ml^-1^). Sampling for proteomic profiling was conducted on day 7, upon which assessment of re-fed cultures began. Time points where data show a statistically significant difference (Student's t-test, n = 3, P<0.05) between Fe-replete and Fe-deplete cultures are indicated by black outer circles for the Fe-replete data points.

Late exponential phase cultures with 120 nM added Fe (~350 pM Fe’) used as innocula for the experimental time series displayed evidence of mild Fe stress (supressed F_v_/F_m_), consistent with previous studies using similar experimental conditions [34]. Subsequently, within the experimental time series F_v_/F_m_ increased rapidly in Fe-replete (Fe+, 400 nM added Fe cultures), which displayed significantly higher F_v_/F_m_ (~0.41–0.54) compared with no added Fe (Fe-, 0 nM added Fe) cultures (0.31–0.41) over the whole duration of the experiment (Student’s t-test, n = 3 P<0.05). Fe+ *Trichodesmium* cultures displayed elevated growth rates (0.43 ± 0.02 d^-1^) compared to Fe stressed cultures (Fe-) (0.36 ± 0.03 d^-1^) (Student's t-test, n = 3 P<0.05), with statistically significant increases in cell density in Fe+ cultures apparent after ~7 days growth (Fig 1). Recovery of growth rates and F_v_/F_m_ following the addition of 400 nM Fe to Fe- cultures after day 7 further confirmed the role of Fe availability in dictating these physiological changes. Observed physiological responses to Fe stress were similar to previous reports [7,26,46].

**Fig 2:**
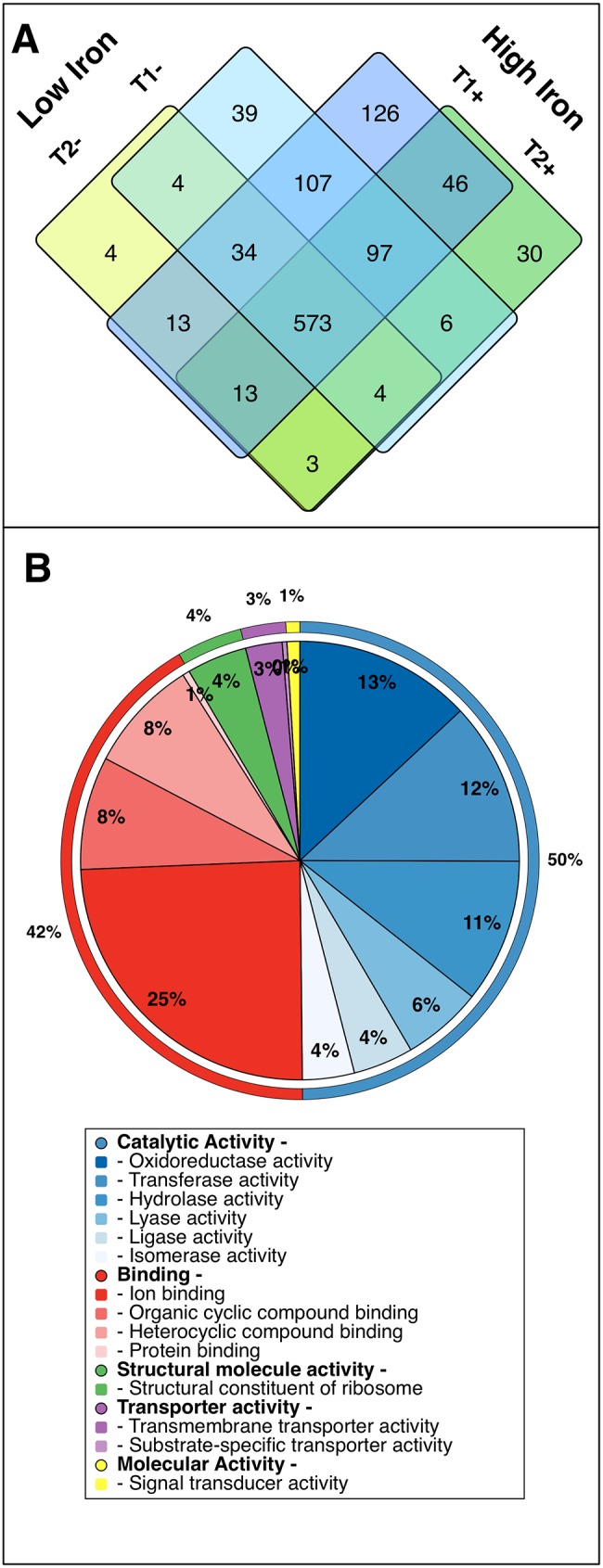
Summary of acquired proteomic data. A) Venn diagram depicting all observed proteins and their distribution amongst our 4 sample treatments (T1+, T1-, T2+ and T2-). B) Pie chart depicting gene ontology terms for all observed proteins as annotated using Blast2GO; external and internal charts show level 2 and 3 cellular component terms, respectively.

**Fig 3:**
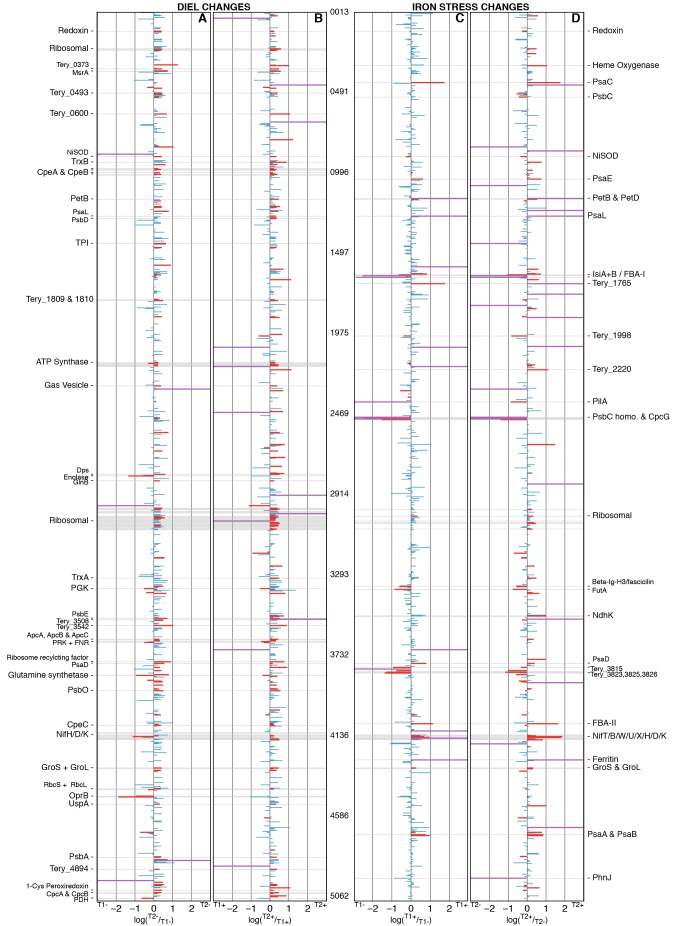
Overview of between-treatment changes in protein abundance. Overview depicting between-treatment changes in protein abundance. Plots A and B show the diel proteomic changes for T1-/T2- and T1+/T2+ respectively. Plots C and D show Fe stress induced proteomic changes for T1-/T1+ and T2-/T2+, respectively. Significant and non-significant changes in protein abundance are colored in red and blue, respectively. The 1104 observed proteins are ordered sequentially along a linear plot of the chromosome and annotated with *T*. *erythraeum* IMS101 locus tags or protein abbreviation/description. Proteins of particular interest are labelled, with some proteins appearing as abbreviations found throughout the text. Proteins for which we observed a statistically significant presence/absence response are plotted as a nominal fold change coloured in purple. Further detail on proteins showing a statistically significant change in abundance can be found in Tables A-D in S1 Data.

Of the 4,451 predicted protein-encoding genes in the *T*. *erythraeum* IMS101 genome (www.jgi.doe.gov), 1104 discrete proteins were observed across all samples using the MS^E^ method. Quantified proteins accounted for 40–80% (average 56%) of the 500 ng of protein loaded on the column with the vast majority of identified spectra confidently identified as *T*. *erythraeum* IMS101 peptides. Remaining unassigned protein may be comprised of peptides incompatible with the proteomic method utilized and/or proteins where post-translational modifications may have prevented annotation to the genome. Individual protein concentrations ranged over 2 orders of magnitude (~2–600 fmol μg^-1^ total protein) with amino acid sequence coverage for all observed proteins ranging from 0.5–95.7% (mean ± σ = 24.7 ± 15.7%), with a minimal sequence coverage for subsequently quantified proteins of 2.9%. The absolute number of observed proteins compares favourably with that of Sandh et al. [22], who detected 1106 potential protein spots using non-quantitative comparative 2-DE/MALDI-TOF-MS in samples of *Trichodesmium* grown with a source of reduced nitrogen. Comparison with Pfreundt et al. [23], who observed 1810 protein-coding transcripts in the *Trichodesmium* primary transcriptome, suggests that our proteome is potentially representative of up to ~60% of expressed genes. Of the 1104 quantified proteins observed here, 573 were observed across all treatments, with 276 only in cultures sampled 2 hours after the onset of the photoperiod (T1+ and T1-) and 37 proteins only in cultures sampled 6 hours after the onset of the photoperiod (T2+ and T2-) (Figs 2 and 3). Forty-seven proteins were exclusively present in Fe-deplete cultures (Fe-) and 202 proteins were exclusively observed in Fe-replete cultures (Fe+) (Fig 2A).

Gene ontology (GO) terms assigned ~92% of the function of identified proteins to catalytic activity (including photosynthesis and N_2_-fixation) or binding (Fig 2B), with oxidoreductase activity and ion binding being the most abundant terms in each parent category. The most abundant proteins were conserved across all 4 treatments and associated with light harvesting, photosynthesis, ATP synthesis and N_2_-fixation (observed at high concentrations ≳100 fmol ug^-1^). Of these, the most abundant proteins were subunits of the phycobilisome light-harvesting complex (concentrations of ~200–600 fmol μg^-1^ total protein, collectively accounting for 9.4–11.3% of the total measured proteome), in agreement with photosynthetic light-harvesting complexes dominating cyanobacterial C and N pools [47].

### Differentially abundant proteins

Statistical analysis identified a total of 210 differentially abundant proteins across the 4 treatments (T1+, T1-, T2+, T2-) (Fig 3). Greatest changes were observed between sampling time points (T1 to T2), indicating significant short-term temporal regulation of metabolic processes (Fig 3). A total of 137 and 109 proteins had significantly different abundances between T1+ and T2+, and T1- and T2-, respectively (Tables A and B in S1 Data). Such a diel change in proteomic composition is consistent with a significant diel cycle in metabolic processes, with T1 and T2 likely representing peak photosynthetic and peak nitrogen fixation rate, respectively [48]. Comparisons between the Fe+ and Fe- proteomes identified fewer differentially abundant proteins, 50 between T1- and T1+, and 111 between T2- and T2+ (Tables C and D in S1 Data). Within the current study of Fe stress physiology we focus on the subset of proteins showing regulation by Fe availability over both sampling time points to identify proteins showing a true Fe stress response rather than changes driven by diel variability.

### Fe-induced changes to the proteome

#### 1. Fe uptake and storage

Deficiency of Fe induced a number of proteomic changes potentially relating to Fe acquisition and storage. *Trichodesmium* is known to utilize inorganic Fe(II) and Fe(III) compounds, along with certain Fe-siderophore complexes [49–52]; however, detailed mechanisms of Fe uptake remain unclear [50,51,53]. FeoAB is an Fe(II) uptake system first characterized in *Escherichia coli* [54]. Although transcripts of FeoB (Tery_2878) have previously been observed to be upregulated in iron-stressed *Trichodesmium* [29], neither protein (FeoA or FeoB) was detected by Sandh et al. [22] nor in any of our samples, potentially suggesting limited importance under the specific Fe conditions analyzed, or that these proteins are not readily detectable with the employed proteomic methods.

**Fig 4:**
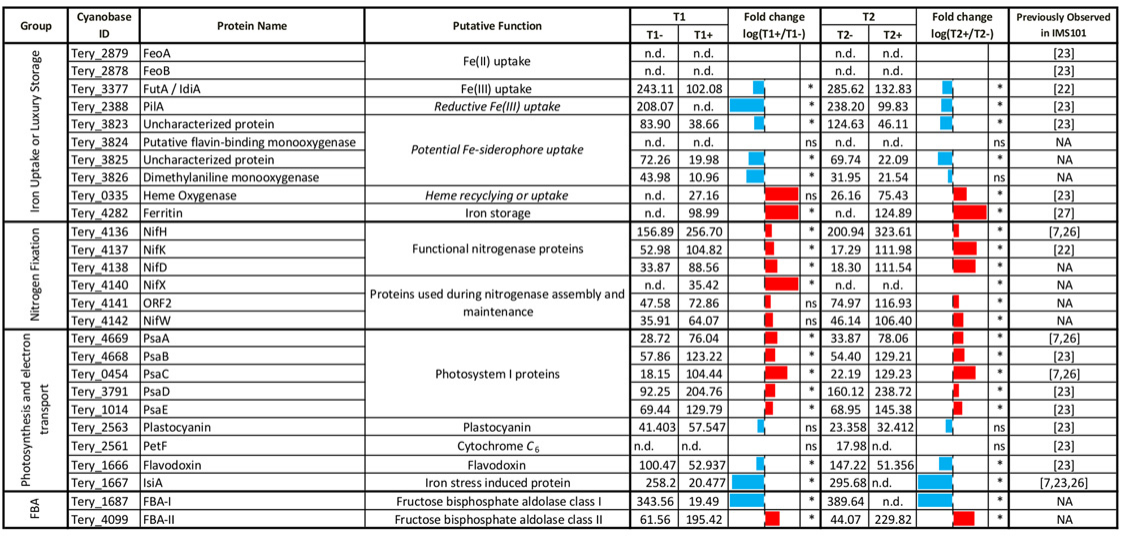
Observed abundance and fold change of selected proteins. All the specifically mentioned proteins alongside their abundances (fmol μg^-1^). Fold changes are shown as sparklines (blue = more abundant during Fe-deplete conditions, red = more abundant during Fe-replete conditions) and statistical significance of change is denoted with an asterisk. The reporting in previous studies is listed in the final column.

*Trichodesmium* is thought to utilise the FutABC system for cytoplasmic Fe(III) membrane transport [29]. Observed elevated concentrations of FutA/IdiA [Tery_3377] (Figs 3 and 4), a protein previously suggested as an environmental Fe stress biomarker [27,55], suggests an increased ability to transport Fe(III) from the periplasm to cytoplasm under Fe-deplete conditions, potentially signifying an increased effort to acquire extracellular Fe.

Fe uptake may also be facilitated through reduction of Fe(III) to the more available Fe(II) by an extracellular or outer-membrane reductive process [51,52,56]. Superoxide-mediated reductive uptake of Fe (III) has recently been proposed for *Trichodesmium* [50]. Increased oxidative stress may also be expected under Fe stress conditions [57]. Within our Fe stressed cultures we observed elevated concentrations of a number of proteins linked to the reduction of oxidative stress through removal of superoxides and other reactive oxygen species. These included nickel superoxide dismutase (NiSOD) [Tery_0971] [58,59], a putative peroxiredoxin (Tery_0162), and two thioredoxins homologous to TrxA and TrxB (Tery_3311 and Tery_0945, respectively). Such observations suggest an increased production of superoxide, which we speculate may be be involved in reductive Fe(III) uptake [50] under Fe stress conditions.

*Trichodesmium spp*. appear to be capable of directly accessing some forms of particulate Fe(III), for example in the form of atmospheric dust [52]. Physical interaction between the cell surface and particulates, alongside extracellular reductive pathway(s) involving the active donation of electrons to insoluble extracellular electron acceptors (such as Fe(III) oxides), may involve structures such as pilins or ‘bacterial nanowires’ [60–62]. For example, Lamb *et al*. [63] recently demonstrated a reduced ability for a *pilA1* mutant (major pilin protein) of the cyanobacteria *Synechocystis sp*. PCC6803 to grow on Fe oxides, suggestive of a role for PilA1 in cyanobacterial reductive Fe uptake. The PilA homolog in *T*. *erythraeum* IMS101 [Tery_2388] was highly abundant in low Fe (T1- and T2-) proteomes and significantly less abundant in high Fe (T1+ and T2+) proteomes (Figs 3 and 4). The observed strong Fe dependence thus suggests a significant role for PilA in the Fe stress response of *Trichodesmium*, potentially linked to reductive Fe uptake [63,64] or motility of particles at the cell surface [52].

A number of the proteins elevated under Fe-deplete conditions were uncharacterized (Tables C and D in S1 Data). Of these, Tery_3823, Tery_3825 and Tery_3826 are of particular interest given their sequential position in the *T*. *erythraeum* IMS101 genome and large increases in abundance under Fe stressed conditions (Figs 3 and 4 and S2 Fig). Tery_3823 is a 162-amino-acid protein, previously observed at the transcript level [23], containing the lipocalin 5-conserved domain (pfam13924). Siderocalins are a sub-group of lipocalins specifically involved in siderophore scavenging, including uptake of the siderophore enterobactin [65], Fe from which is released by ferric enterobactin esterase [66]. No homolog of ferric enterobactin esterase is present in the *T*. *erythraeum* IMS101 genome but Tery_3825 contains a putative lipase/esterase conserved domain (pfam01764) (S2 Fig). Tery_3824 (observed but not Fe regulated) contains a TrkA conserved domain predicted to be involved in inorganic ion transport (COG2072), whilst Tery_3826 has a conserved flavin-binding monooxygenase (pfam13738) known to be involved in siderophore production [67] (S2 Fig). Although not thought to produce siderophores, *Trichodesmium* colonies have been shown to be capable of Fe acquisition from externally derived siderophores [49,51]. Considering that *T*. *erythraeum* IMS101 has no known TonB-dependent outer-membrane receptors [29,68], we suggest that the Fe-regulated cluster of proteins Tery_3823–3826 may be related to an unconventional Fe stress-induced siderophore uptake system. In contrast, the periplasmic ferric siderophore-binding protein FhuD (Tery_3943), thought to be involved in the uptake of ferrichrome and other hydroxamate siderophores [69,70], was observed at low abundances across all conditions.

Heme oxygenase (HO, Tery_0335), responsible for catalyzing the degradation of heme and the subsequent release of Fe, was observed at elevated Fe availability, particularly within sample T2+ (Fig 3) when N_2_-fixation was likely higher [16]. The presence of HO may indicate a degree of intracellular Fe re-purposing such as proposed for the elevated HO observed during the active N_2_-fixing period of *Crocosphaera watsonii* [37]. Alternatively, HO localization in the outer membrane as a mechanism for exogenous heme uptake has been well studied in pathogenic bacteria [71] and a few marine bacterial isolates [72–75].

Lastly, the Fe-storage protein ferritin (Tery_4282) was abundant under Fe-replete conditions, as previously observed [22], but entirely absent under Fe-deficient conditions (Figs 3 & 4). Ferritin facilitates luxury uptake and storage under Fe-replete conditions, generating reserves that are presumably available to the cell under Fe stress. The absence of ferritin under Fe stress thus suggests that proteomic Fe was dominated by functional pools under these Fe-depleted conditions.

#### 2. Metabolism

The data-independent method, MS^E^, provides high confidence in the quantification of individual proteins and allows for the interrogation of the internal stoichiometry of multi-subunit complexes such as nitrogenase and the photosynthetic catalysts (S3 Fig). In the majority of cases, subunit complexes demonstrated consistency with predictions based on known structures and associated subunit stoichiometry (Table 1 and S3 Fig). Occasionally, this approach also revealed potentially interesting deviations in subunit:complex ratios which may warrant further investigation. One such observation was the consistent 2:1 ratio of PsbO:PSII. PsbO is involved in stabilization of the catalytic site in photosystem II (PSII) and the crystal structure of cyanobacterial PSII suggests a PsbO:PSII stoichiometry of 1:1 [76,77]. However, experimental evidence suggests a 2:1 stoichiometry in higher plants [78,79], with the different PsbO isoforms enabling increased stabilization of the PSII catalytic site. Our data may suggest an as-yet-unconsidered role of PsbO *in vivo* in cyanobacterial species.

**Table 1:** Average concentration of select multi-subunit protein complexes.

Complex	T1+	T1-	T2+	T2-
**PSII**	85.0 ± 13.0	112.4 ± 17.2	131.6 ± 19.8	148.3 ± 21.8
**Cytochrome b**_**6**_ **f**	69.7 ± 14.9 *(0*.*8*:*1)*	56.3 ± 14.2 *(0*.*5*:*1)*	84.0 ± 24.2 *(0*.*6*:*1)*	71.5 ± 17.5 *(0*.*5*:*1)*
**PSI**	134.0 ± 16.0 *(1*.*6*:*1)*	56.3 ± 12.8 *(0*.*5*:*1)*	156.1 ± 22.5 *(1*.*2*:*1)*	74.4 ± 24.9 *(0*.*5*:*1)*
**RuBisCO**	224.1 ± 30.1 *(2*.*6*:*1)*	255.4 ± 113.44 *(2*.*3*:*1)*	224.0 ± 46.8 *(1*.*7*:*1)*	250.9 ± 84.8 *(1*.*7*:*1)*
**ATP Synthase**	97.4 ± 22.0 *(1*.*1*:*1)*	113.9 ± 33.1 *(1*.*0*:*1)*	144.7 ± 35.2 *(1*.*1*:*1)*	134.3 ± 41.9 *(0*.*9*:*1)*
**Nitrogenase**	81.5 ± 15.8 *(1*.*0*:*1)*	39.4 ± 6.8 *(0*.*4*:*1)*	96 ± 15.6 *(0*.*7*:*1)*	25.2 ± 7.4 *(0*.*2*:*1)*
**IsiA**	20.5 *(0*.*2*:*1)*	258.2 *(2*.*3*:*1)*	n.d. *(NA*)	295.7 *(2*.*0*:*1)*

Average concentration ± standard error (fmol.μg^-1^ total protein) for select multi-subunit protein complexes observed across each of the 4 samples. Bracketed and in italic font are the complex:PSII ratios.

Generally, the largest decreases observed under reduced Fe availability were associated with those proteins known to contain Fe cofactors, the most significant of which were the Fe-containing components of nitrogenase NifH, NifD and NifK (Tery_4136, 4137 and 4138, respectively) (Figs 3 & 4). Concentrations of the whole nitrogenase complex were therefore significantly reduced under Fe stress (Student’s t-test, P<0.05), with complex abundances for Fe- samples ~2-3-fold lower than for Fe+ samples (Table 1). Such a reduction in Fe-rich functional nitrogenase proteins NifD/K and NifH is a well-documented Fe compensation response, having been observed at both the transcriptional [26] and protein levels [7], as well as being consistent with observed nitrogen fixation rates [20]. Corresponding decreases in ancillary nitrogenase proteins NifX [Tery_4140], ORF2 [Tery_4141] and NifW [Tery_4142] were also observed (Fig 3 and S1 File).

The abundances of numerous proteins involved in photosynthetic electron transport also decreased under Fe stress. Largest changes were observed for subunits of the Fe-rich photosystem I (PSI) complex, including PsaA (4 Fe, Tery_4669), PsaB (Tery_4668), PsaC (8 Fe, Tery_0454), PsaD (Tery_3791) and PsaE (Tery_1014) (Fig 3) indicating a significant decrease in overall PSI concentrations (Table 1). Reduction of the Fe-rich PSI complex in the absence of similar changes in photosystem II (PSII) concentrations resulted in a marked shift in PSI:PSII stoichiometry, decreasing from ~1.2–1.6:1 under Fe-replete growth conditions to ~0.5:1 under Fe-deplete growth conditions (Table 1). Such changes are in agreement with previous transcriptional studies [26] and antibody-based quantifications of *Trichodesmium* PSI:PSII ratios under Fe stress [7,21] as well as being broadly consistent with observations from non-diazatrophic cyanobacteria [80,81]. Reductions in the number of complete PSI complexes were also accompanied by increases in the abundance of the chlorophyll-binding Fe stress-induced protein IsiA [Tery_1667], which was ~13-fold more abundant in low Fe samples (Fig 3 and Table 1) resulting in an IsiA:PSI ratio of 4–4.5:1 under Fe-stress. IsiA has been proposed to increase the absorption cross-section of PSI as a compensation strategy to mitigate reductions in PSI reaction centre concentration [81–84] through the formation of a IsiA-PSI supercomplex having an ~6:1 IsiA:PSI ratio. IsiA may also fulfil additional roles in photoprotection and/or as a chlorophyll store [81–84]. Such multiple functions may explain why measured IsiA:PSI ratios can exceed 6:1 under Fe stress in cyanobacteria [80,81]. Within the current study, the observed <6:1 ratio of IsiA:PSI is consistent with previous reports from culture and field-studies [7] potentially suggesting that *Trichodesmium* does not accumulate significant surplus amounts of IsiA under iron-stress [80,85]. Irrespective of the specific role of IsiA, elevated concentrations under Fe stress confirm its role in a Fe-efficient photosynthetic strategy.

**Fig 5:**
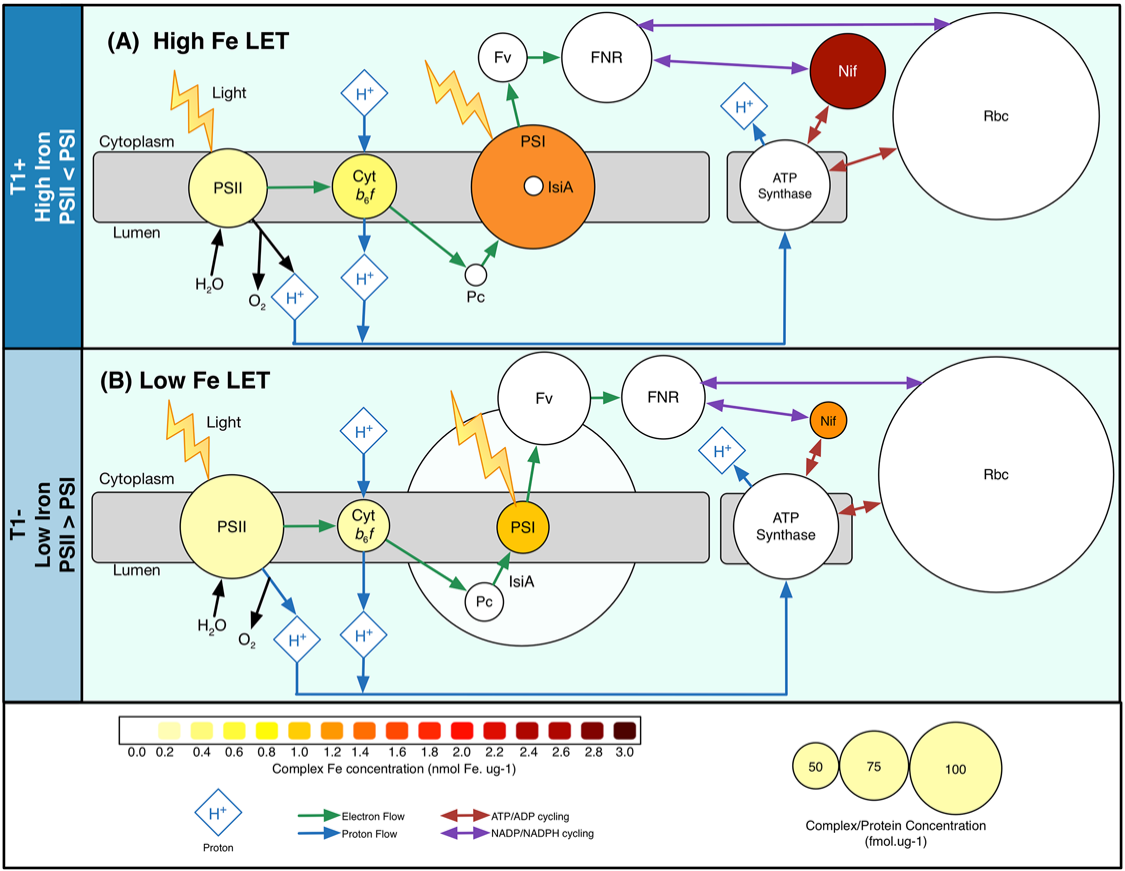
Linear electron flow schematic during high and low Fe conditions. Simplified schematic demonstrating the linear electron transport pathway during both high Fe (A, T1+) and low Fe (B, T1-) conditions. Protein complexes are shown as circles with their diameter indicative of observed complex concentration. Circles are coloured so as to show the predicted Fe concentration of that protein complex. Abbreviations include–PSII–photosystem II, Cyt b_6_f –Cytochrome b_6_f, PSI–photosystem I, Pc–plastocyanin, IsiA–iron stress induced protein A, Fv–Flavodoxin, FNR–Ferredoxin-NADP reductase, Rbc–RuBisCO, Nif–nitrogenase, ATP synthase–adenosine triphosphate synthase.

Although the abundance of PSI was significantly reduced under Fe stress, no equivalent reductions in the abundance of the Fe-rich Cytochrome *b*_6_ *f* (Cyt- *b*_6_ *f*) complex were observed, as reflected in the minimal Fe-stress induced changes in Cyt-b_6_f:PSII ratios (Table 1) which were in line with previous observations of Fe-stressed cyanobacteria [80]. Hence PSI:Cyt-*b*_6_ *f* reduced from ~2:1 under Fe-replete conditions to 1:1 under Fe-deplete conditions (Table 1 and Fig 5), potentially linked to the dual role of the Cyt-*b*_6_ *f* complex in respiratory and photosynthetic electron transfer in cyanobacterial cells [86].

Flavodoxin [Tery_1666], the Fe-free functional replacement of the soluble electron carrier ferredoxin, was present in all proteomes and demonstrated a significant increase in abundance under Fe stress conditions (Fig 5), a well-documented Fe compensation strategy [27,28]. Ferredoxin was not observed in any treatment and is potentially not detectible using the employed MS^E^ method. However we note that the concentration of ferredoxin-NADP reductase (FNR, [Tery_3658]), which transports electrons from ferredoxin/flavodoxin to nicotinamide adenine dinucleotide phosphate (NADPH), was similar between treatments (Fig 5).

Fructose bisphosphate aldolase (FBA) enzymes also displayed highly significant Fe stress responses in *T*. *erythraeum* IMS101, with pairwise substitution of the class II FBA [Tery_4099], which was abundant under Fe-replete conditions, for a class I FBA [Tery_1687] under Fe stress. Similar substitutions have been observed in diatoms under Fe stress conditions [87,88]. Class II FBAs are dependent on a divalent cation such as Zn^2+^ or Fe^2+^ [89,90]. Although Tery_4099 is most similar to the Co^2+^-containing FBA of *Thermus aquaticus* [91], it appears that replacement of class II with class I FBAs may be a common response to Fe stress [88]. Irrespectively, ratios of Tery_1687/Tery_4099 may be a good candidate marker of Fe stress within field populations of *Trichodesmium*. Indeed, changes in both FBA I or II concentrations were amongst the largest observed under Fe stress, suggesting that FBAI/II ratios may be a more sensitive and reliable biomarker than, for example, flavodoxin.

### Proteomic Fe Allocation

**Fig 6:**
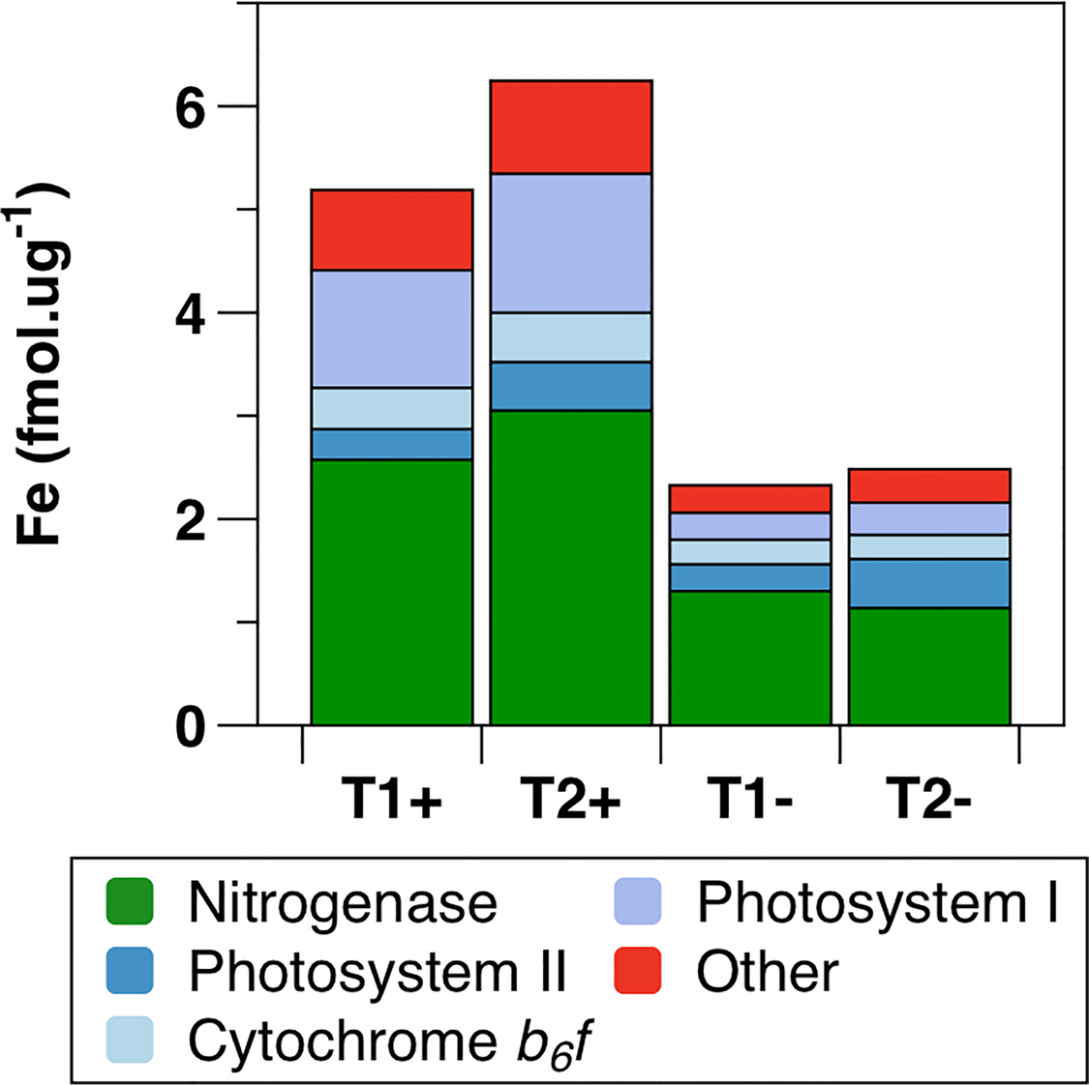
Total predicted protein associated Fe. Total protein derived Fe concentration for each of the four experimental treatments (T1+, T2+, T1- and T2-) expressed as fmol Fe ug^-1^ total protein. Fe concentration is subdivided into the major Fe containing complexes: Nitrogenase, cytochrome *b*_6_ *f*, PSII, PSI and other.

The applied proteomic technique enables estimation of absolute protein concentrations [30,31], facilitating estimation of relative metabolic cellular Fe quotas for each sample (see Methods). Of the 1104 total observed proteins, 43 were predicted to be Fe-binding. Concentrations of Fe associated with these proteins (fmol Fe μg^-1^ total protein) were derived from the product of protein abundance and known or predicted Fe binding stoichiometries. A full sampling of the complete Fe-binding proteome cannot be guaranteed using the MS^E^ proteomic technique. However, consideration of the proportional contributions of observed Fe-binding proteins as a function of protein concentration suggests that any under-sampled low abundance proteins likely contributed little to the overall Fe-binding proteome (S4 Fig). A significant decrease in total protein-associated Fe was observed within both the Fe-deplete proteomes, with T1- and T2- predicted to contain 55% and 60% less protein-bound Fe than their Fe-replete counterparts (Fig 6). Consistent with previous reports [7,92], the largest pool of metabolic intracellular Fe in *T*. *erythraeum* IMS101 was estimated to be associated with the multi-subunit nitrogenase complex, with 46–56% and 49–50% of the predicted protein-bound Fe associated with this complex under Fe-deplete and Fe-replete conditions, respectively (Fig 6). The various Fe-binding components of the photosynthetic electron transport chain (PSII, Cyt-*b*_6_ *f* and PSI cumulatively) accounted for a further 33–41% of total proteomic Fe, with the largest share of this attributed to PSI (11–13% Fe-deplete, 22% Fe-replete) (Fig 6). In addition to Fe bound within metabolic complexes, Fe storage within ferritin was likely significant under high Fe conditions. Quantification of the Fe associated with ferritin is complicated by the potential variable loading of ferrihydrite-phosphate within the core of the multimeric complex [93]. However, if, for example, we assume a stoichiometry of ~260 Fe:protein [93], we would estimate that up to 84% of the total cellular Fe might be stored within ferritin under Fe-replete conditions.

**Fig 7:**
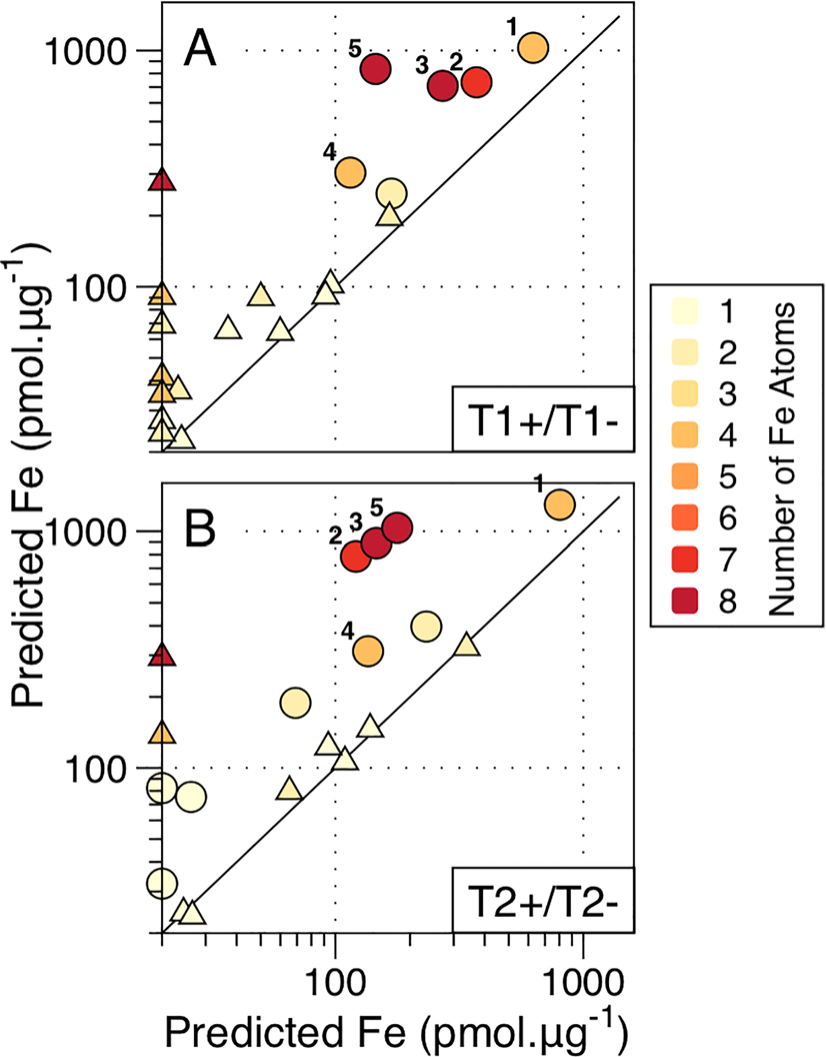
Predicted protein Fe concentrations. Scatter plots showing predicted Fe concentration per total protein for those proteins thought to contain one or more Fe cofactors as derived from bioinformatical method described in the text and the quantified protein concentrations observed. Comparisons are shown for T1+/T1- and T2+/T2-. Ferritin has been omitted due to the potential variable Fe:protein ratio discussed in the text. Shade denotes the predicted number of Fe atoms per protein. Proteins showing a statistically significant difference between treatments are shown as circles whilst proteins having non-significant differences are shown as triangles. Select proteins are labelled: NifH (1), NifD (2), NifK (3), PsaA (4) PsaC (5). Note logarithmic scale. Also note that statistical significance was determined from observed protein concentrations and not predicted Fe concentrations. Consequently in some cases non-statistically significant changes in observed protein concentrations could still represent substantial differences in overall protein-bound Fe where proteins have high predicted Fe-binding stoichiometries.

Further statistical analysis demonstrated that the overall reduction in non-ferritin proteomic Fe following development of Fe stress was predominantly associated with proteins containing >2 Fe atoms within iron-sulphur (FeS) complexes (Fig 7). Conversely, heme-containing proteins such as those of Cyt-*b*_6_ *f* or the PsbE/ Cyt *b*_559_ of PSII, were less impacted by Fe stress. Proteins with the largest individual Fe requirements hence appear to be preferentially sacrificed in response to Fe stress [26]. Such a strategy would be expected to result in the minimum potential alteration of the overall proteome for a given decrease in metabolic Fe. Thus, although significant impacts on metabolic processes associated with preferentially lost proteins are likely [26], *Trichodesmium* might reasonably be expected to achieve a degree of minimization of overall metabolic impacts for a given Fe saving through preferential decreases in the most Fe-rich proteins.

### Potential implications for alternative photosynthetic strategy under Fe stress

Estimates of resource allocation to components of the photosynthetic electron transport chain enables consideration of potential changes in overall photosynthetic strategy [94]. Oxygenic photosynthesis produces both reductant, as the electron carrier NADPH, and energy, as adenosine triphosphate (ATP) (Fig 5). Linear photosynthetic pathways (Linear electron flow, LEF), where electrons flow from PSII to Cyt-*b*_6_ *f* to PSI, result in a relatively high NADPH to ATP ratio [94,95]. However, the overall ATP demand in cells is typically greater than NADPH demand so a variety of alternative photosynthetic pathways (Alternative electron flow, AEF) cycle electrons round PSI and transfer electrons from PSII to water via midstream or terminal oxidases [94,96], resulting in enhanced ATP to NADPH generation.

AEF pathways are likely to be particularly important in diazotrophs, which require additional ATP for N_2_-fixation (>16 ATP per N_2_ fixed)[97]. Predicted Fe demands and PSII:PSI ratios associated with maintenance of different LEF and AEF pathways [94,96] suggest that AEF involving PSII and increased PSII>PSI can result in a more efficient use of Fe to generate ATP. Under Fe-replete conditions our proteomic data suggest a potential excess of PSI and hence maximum PSI capacities may exceed maximum PSII capacities (Table 1 and Fig 5), suggestive of a reliance on (cyclic or pseudocyclic) electron cycling around PSI to generate additional ATP [16,18]. Use of AEF around PSI potentially reduces oxygen evolution and formation of reactive oxygen species from PSII, which can be detrimental to nitrogenase and may even contribute to lowering cellular O_2_ through a pseudocyclic pathway [18]. However, substantial Fe may be required [94,96].

Conversely, under Fe stress, maximum PSII capacities may exceed maximum PSI capacities, significantly reducing Fe demands for additional ATP production [94]. Increases in PSII:PSI under Fe stress (Table 1) might necessitate increased scavenging of molecular oxygen or superoxide generated by PSII to prevent nitrogenase inhibition [18,22] for example using aforementioned NiSOD, peroxiredoxin, TrxA and TrxB. While cyclic PSI electron flow is likely still important, the observed reduction of PSI:PSII, from ~1.2–1.6 during Fe-replete conditions to ~0.5 during Fe-deplete conditions, may suggest increased reliance on AEF pathways associated with PSII-catalyzed water-water cycles [94]. Midstream oxidase (PSII-MOX) or PSII respiratory terminal oxidase (PSII-RTO) are thought to be required to facilitate these AEF pathways. Although the *T*. *erythraeum* IMS101 genome codes for two RTOs, cytochrome *c* oxidase (COX, Tery_1777–9) and an alternative respiratory terminal oxidase (ARTO, Tery_0276–8), neither could be observed, suggesting they were either below detectible abundances or were not readily detectable using the adopted proteomic method. Speculatively, transfer of electrons to the cell surface to facilitate reductive Fe uptake [53,56,63] could form an alternate sink for a small proportion of the surplus electrons derived from PSII under Fe stress.

## Conclusion

The results presented describe holistic, quantified proteomic changes in *Trichodesmium* in response to reduced Fe availability. The observed proteomic plasticity indicates how co-occurring processes simultaneously act to alleviate Fe deficiency through extracellular uptake and intracellular recycling/repurposing processes. Further, a general decrease in abundance, and therefore the maximum enzymatic capacities, of the photosynthetic and N_2_-fixing apparatus suggested a general Fe retrenchment response under conditions where *Trichodesmium* IMS101 had exhausted acquisitional and compensatory adaptations to Fe deficiency. In addition, the radical remodelling of the photosynthetic electron transfer chain suggests the potential for alternative Fe-efficient electron-flow pathways under Fe stress conditions. Remodelling of core physiological processes resulted in a proteome predicted to require ~50% less metabolic Fe, driven in most part by reduction of Fe-rich proteins, with subsequent impact on the elemental and macromolecular composition of *Trichodesmium* which would have significant consequences for biogeochemical cycles [7,92,98]. When considered in the context of intermittent and spatially variable Fe inputs to the surface ocean [99–101], the observed multi-faceted proteomic response to Fe stress, involving storage, acquisition and compensation strategies, may afford *Trichodesmium* the ability to readily reduce their proteomic Fe burden whilst maintaining core physiological processes while awaiting more favourable growth conditions.

## Supporting Information

S1 Data Data tables A (T1- vs T1+), B (T2- vs T2+), C (T1- vs T2-) and D (T1+ vs T2+) present all proteins observed to undergo a statistically significant (P<0.05) change in abundance, as determined by two-way ANOVA with a Bonferroni correction. Uniprot Accession and Tery no.'s (as referred to in the text) are displayed alongside protein description sourced from the Uniprot database. For each treatment (T1-, T1+, T2- and T2+) the mean concentration (fmol.ug^-1^) and standard deviation of the three biological replicates are displayed alongside the multiple comparison adjusted P value. An instance where a protein was not observed in any one treatment is denoted by 'n.d.'. Data table E contains non-biomass normalized protein concentration data for each of the technical and biological replicates across the 4 treatments. Data is shown as 'ng on column' and 'fmol on column' values for each of the 18 analytical runs are per experimental condition (T1-, T1+, T2-, T2+). Data was processed as detailed in the methods section prior to statistical analysis.
(XLSX)

S1 Fig Cumulative protein concentration for each of the 4 analysed proteomes: T1- (light blue), T1+ (dark blue), T2- (light green), T2+ (dark green). All 3 biological replicates are indicated as individual lines. The two excluded biological replicates– T1-_3 and T2-_1 are indicated in with dashed light blue and light green lines respectively. The number of proteins which together comprise 95% of the observed proteome is indicated by solid vertical lines and equates to T1- = 574, T1+ = 632, T2- = 434 and T2+ = 483.
(TIF)

S2 Fig Gene organisation (green) of Tery_3823, 3824, 3825 and 3836 in the *T*. *erythraeum* IMS101’s genome. Identified conserved domains are indicated in black and discussed in the main text.
(TIF)

S3 Fig Relative stoichiometric ratios of select multi-protein complexes compared with literature values. Stoichiometries are calculated from the concentration of a given protein divided by the average concentration of the complete multi-protein complex. Literature derived ‘predicted’ stoichiometric ratios are shown in red.
(TIF)

S4 Fig Bar chart illustrating the diminishing contribution of less abundant proteins to the total predicted Fe proteome. Data displayed is for the percentage each Fe containing protein contributes to the total proteomic Fe pool. Treatments are shown as: T1- (light blue), T1+ (dark blue), T2- (light green), T2+ (dark green).
(EPS)

S1 File Supplementary discussion comparing literature values for stoichiometric ratios of known multi-protein complexes compared to those observed in our study. (DOCX)
