# Supplementary figures and images for: Quantifying Integrated Proteomic Responses to Iron Stress in the Globally Important Marine Diazotroph Trichodesmium
Source: PLoS One. 2015 Nov 12;10(11):e0142626. doi: 10.1371/journal.pone.0142626 (PMC4642986; doi:10.1371/journal.pone.0142626)

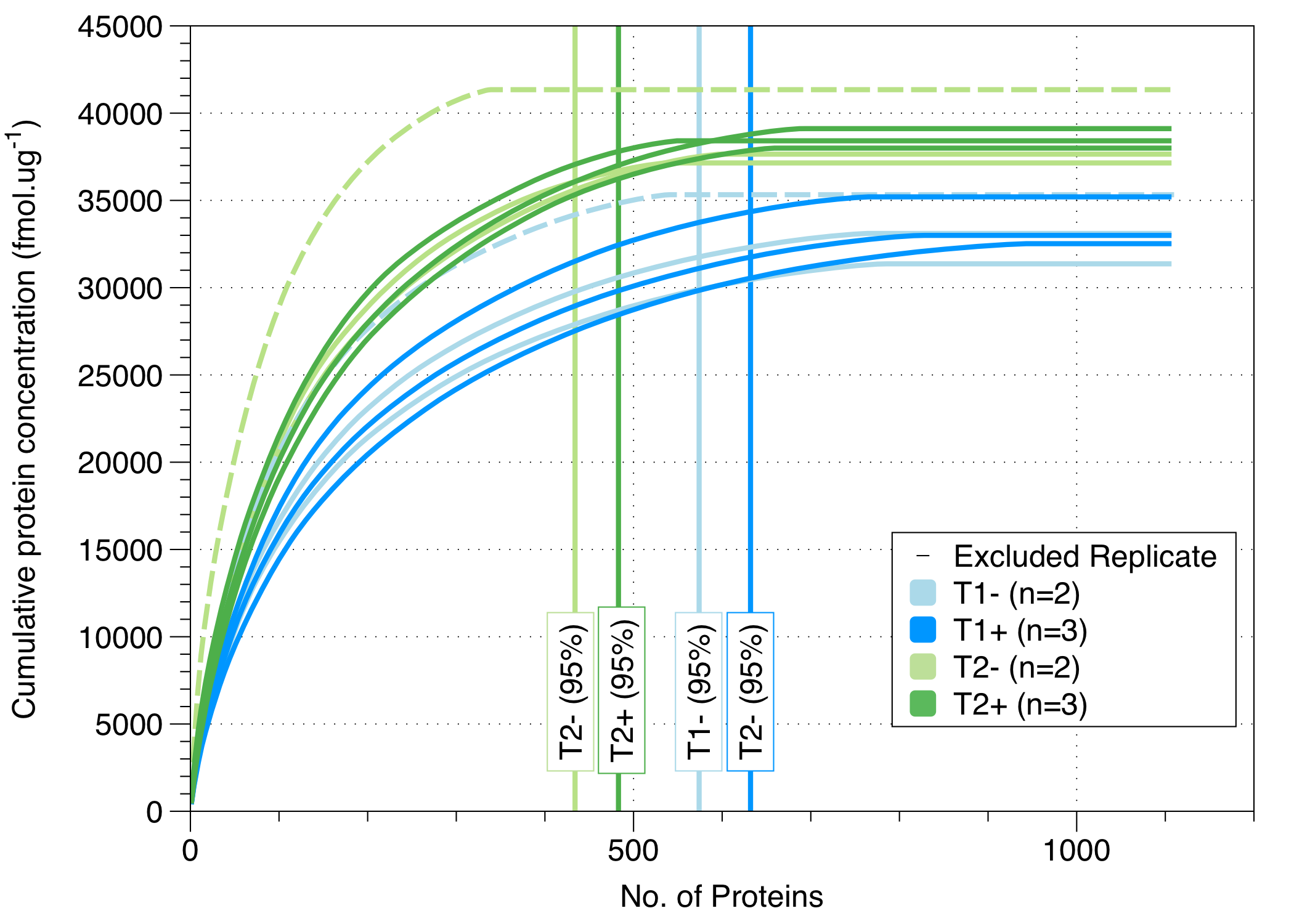

Supplement: S1 Fig — All 3 biological replicates are indicated as individual lines. The two excluded biological replicates– T1-_3 and T2-_1 are indicated in with dashed light blue and light green lines respectively. The number of proteins which together comprise 95% of the observed proteome is indicated by solid vertical lines and equates to T1- = 574, T1+ = 632, T2- = 434 and T2+ = 483. (TIF) [file pone.0142626.s002.tif]

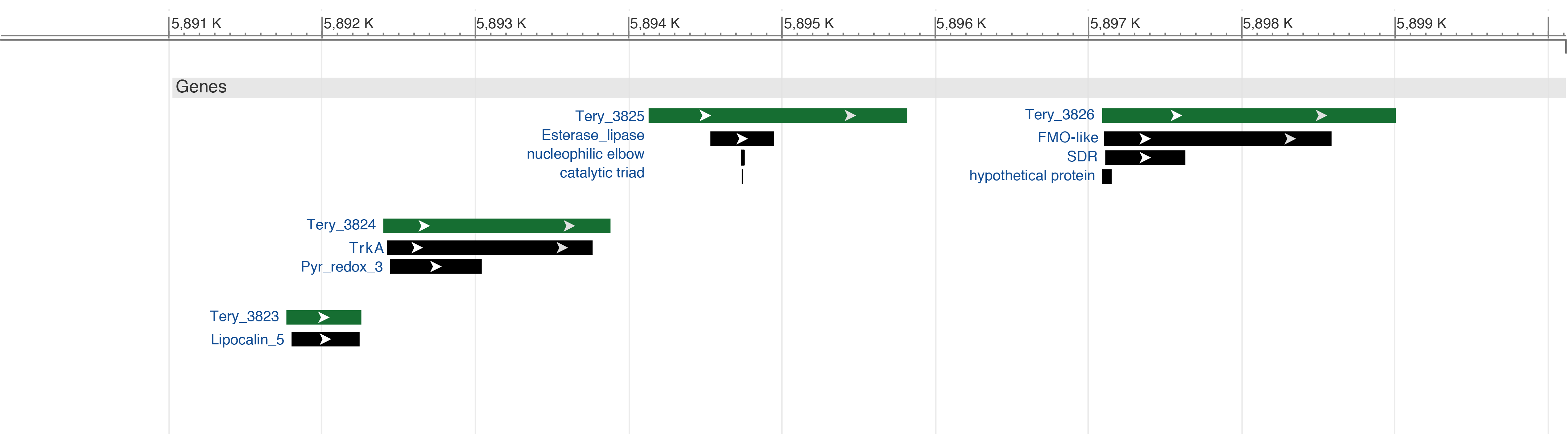

Supplement: S2 Fig — Identified conserved domains are indicated in black and discussed in the main text. (TIF) [file pone.0142626.s003.tif]

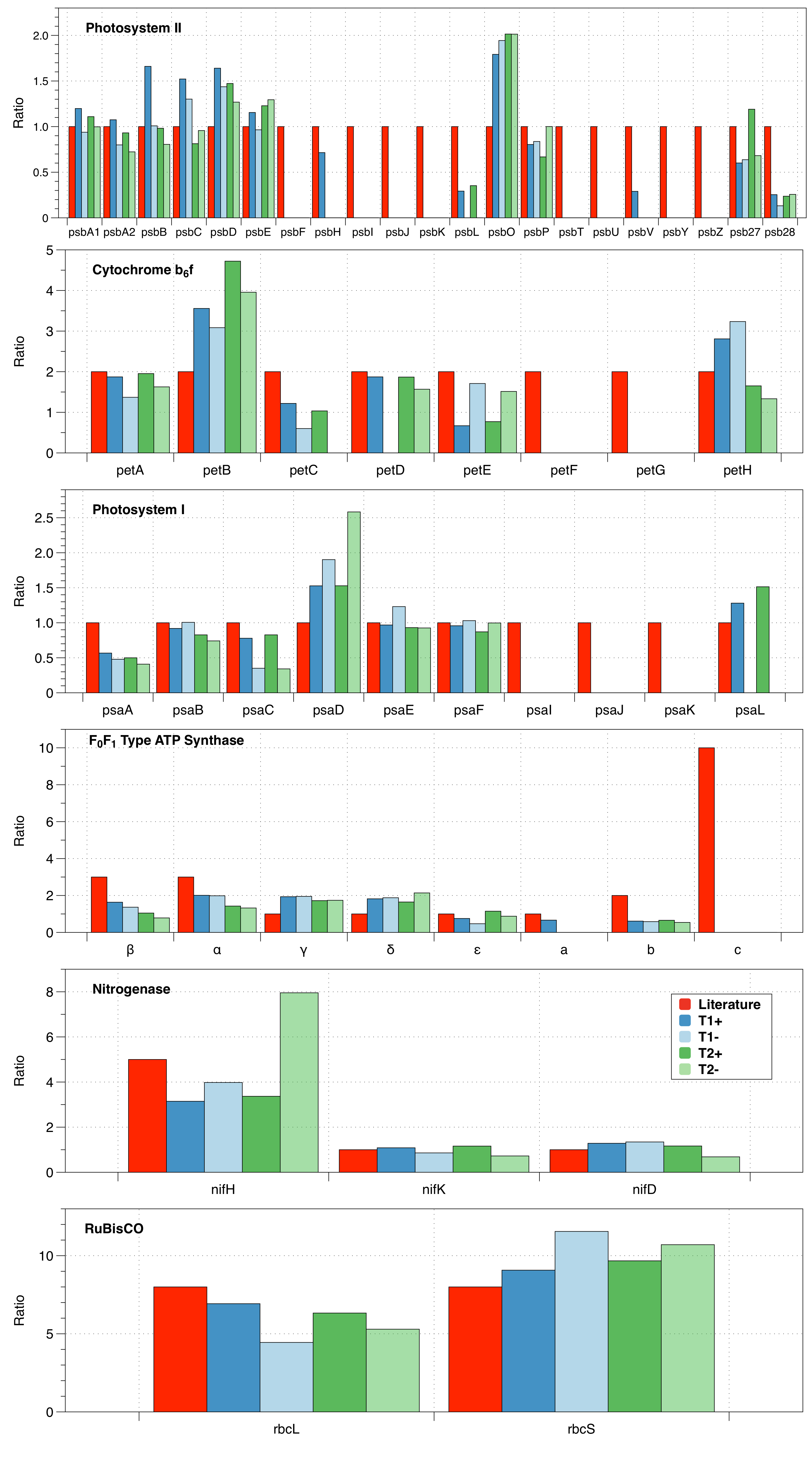

Supplement: S3 Fig — Stoichiometries are calculated from the concentration of a given protein divided by the average concentration of the complete multi-protein complex. Literature derived ‘predicted’ stoichiometric ratios are shown in red. (TIF) [file pone.0142626.s004.tif]
