# Supplementary material for: Quantifying Integrated Proteomic Responses to Iron Stress in the Globally Important Marine Diazotroph Trichodesmium
Source: PLoS One. 2015 Nov 12;10(11):e0142626. doi: 10.1371/journal.pone.0142626 (PMC4642986; doi:10.1371/journal.pone.0142626)
Supplement: S1 File — (DOCX) [file pone.0142626.s006.docx]

# Supplementary Information

## Multi-submit protein complexes

Literature values for stoichiometric ratios of known multi-protein complexes were compared to our results in order to provide a means of internal data validation. As published stoichiometric ratios are often derived from isolated functional complexes they may not necessarily be indicative of subunit ratios *in vivo* as presented here. However, where the majority of subunits agree with published stoichiometry we view deviations as possibly warranting further investigation. Data are presented concerning the multi-subunit complexes PSII, cytochrome *b*_6_*f*, PSI, F_0_F_1_ type ATP-synthase, nitrogenase and RuBisCO for which stoichiometrically corrected complex concentrations are detailed in the main text (Table 1).

PSII

Cyanobacterial PSII comprises 20 subunits of which 13 were observed in this study, this included the majority of the intrinsic core subunits, PsbA - D1, PsbB - CP47, PsbC - CP43, PsbD - D2, PsbE - α-subunit of cytochrome *b*_559_, PsbF - β-subunit of cytochrome *b*_559_ and finally PsbI [1] (S3 Fig.), most of which were observed at, or near, the expected 1:1 ratio. Of the extrinsic proteins PsbO, PsbP, PsbU and PsbV, only PsbO and PsbP were observed.

Of note are the two distinct isoforms of PsbA at near equimolar concentration and conserved PsbA1/2: PSII stoichiometry (where the concentration of PSII is the average concentration of all observed PSII proteins). The Mn-containing oxygen-evolving complex (OEC) of PSII is located on the lumenal surface of the PsbA/D1 protein and is stabilised by PsbO [2]. As discussed in the main text, we observed a 2:1 PsbO:PSII stoichiometry. Given our observations of two PsbA isoforms, PsbA1 and PsbA2, along with a stoichiometric equivalent of 2 PsbO proteins to each complete PSII complex, it is possible that diazotrophic PSII includes two interchangeable PsbA-OEC-PsbO complexes. Neither PsbA1 nor 2 demonstrated significant time-of-day variability, suggesting both proteins are conserved across the diel cycle. Whilst one PsbA-OEC-PsbO complex will be fully functional and readily evolve oxygen, the second may be a non-oxygen evolving isoform utilized during diazotrophic growth and used to maintain the structural integrity of the PSII super-complex and resist oxidative damage [3,4]. Murray [3] previously determined that no such "rogue-D1" was present in the *T. erythraeum* IMS101 genome, and concluded that the structural differences between PsbA1 and 2 were not sufficient to attribute them with differing functions. Our observations of a ~1:1 ratios for the two isoforms perhaps suggests an alternative strategy than that proposed by Murray [3] .

Most of the 7 PSII proteins unobserved in this study have been observed at the transcriptional level in *Trichodesmium* [5]. However, to the best of our knowledge they have not been observed at the proteomic level, suggesting they are incompatible with mass spectrometry-based techniques or are not readily identifiable using the *T.* *erythraeum* IMS101 genome, potentially due to post translation modification.

Cytochrome b_6_f

Cytochrome *b*_6_*f* is a dimeric protein complex forming a major component of the photosynthetic electron transport chain and aiding in the transfer of soluble electron carriers between PSII and PSI. Cytochrome *b*_6_*f* contains 8 tightly bound polypeptide subunits [6] all present in a 2:2 stoichiometry. Of these 8 subunits, Cytochrome *f* (PetA) and the Rieske Fe-sulphur protein (PetC) are both membrane extrinsic. All membrane-intrinsic and extrinsic proteins are observed with the exception of PetF and PetG. Cytochrome *f* (1 Fe, PetA), cytochrome *b*_6_ (2 Fe, PetB) and the Rieske protein (1 Fe, PetC) all show relative reductions in stoichiometry during Fe-deplete conditions (S3 Fig.); however, the absolute concentration of cytochrome *b*6 (PetB) shows unexplained elevation relative to all other observed sub-units (S3 Fig.) which may warrant further investigation.

PSI

Cyanobacterial PSI contains 7 intrinsic proteins (PsaA, PsaB, PsaF, PsaI, PsaJ, PsaK and PsaL) and a further 3 extrinsic proteins (PsaC, PsaD and PsaE) (KEGG:ter00195) [7,8]. Many PSI subunits are present at a near 1:1 ratio similar to that observed for the isolated complex in the literature [9]. PsaD is present in a >1:1 ratio. PsaD, alongside PsaC and PsaE, is involved in the formation of the ferredoxin /flavodoxin docking site. The ratios of PsaD:PSI is closer to 1.5:1 in samples T1+, T1- and T2+ and closer to 2.5:1 in sample T2-. The Fa and Fb Fe4S4 containing protein PsaC decreases in abundance relative to PSI in response to Fe deficiency. The remaining Fe4S4 cluster, Fx, is liganded between PsaA and PsaB. PsaA displays a smaller Fe stress decrease to PsaC but is detected at a <1:1 ratio.

F_0_F_1_ type ATP Synthase

We did not observe membrane-bound subunits a, b and c, whilst non-membrane proteins α and β were observed to be deplete relative to the membrane-extrinsic proteins ϒ and δ for chloroplastic F_0_F_1_ type ATP-synthase of α_3_β_3_ϒδab_2_c_10-14._ Therefore our results did not show the expected stoichiometry for this complex, a finding that warrants further investigation.

Nitrogenase

The ratio of the heterodimeric proteins NifK:NifD of 2:2 is well conserved across all treatments. However, the relative abundance of NifH to both NifD and NifK shows a distinct Fe stress response; Fe-replete samples, T1+ and T2+, are present at ~3:1 whilst Fe-deplete cultures show ratios of ~4:1 to ~8:1 for T1- and T2-, respectively. This change was predominantly driven by a decrease in the abundance of Fe-rich NifK and NifD relative to NifH. Given the theoretically optimal configuration of NifH:NifDK of 5:1 [10], our observations of an increased NifH:NifDK ratio under Fe stress may be an Fe compensatory response where Fe stress forces a more Fe-efficient nitrogenase configuration.

In addition to the reduction in nitrogenase complex abundance (discussed in the main text, Table 1) are the observed decreases in a number of ancillary nitrogenase proteins including NifX, ORF2 and NifW (Tery_4140, 4141 and 4142, respectively) under Fe stress. The function of NifW is unclear with scant evidence suggesting a role in protecting NifD from oxidative damage [11]. While a concomitant decrease in abundance of the functional nitrogenase proteins is observed, the ratio of NifW to both NifD and NifK varies with Fe treatment. During the later sampling period, the Fe-deplete culture (T2-) had a NifW:NifD/K ratio of ~2 whilst the Fe-replete sample (T2+) had a ratio of ~1, a similar change in ratio was not however observed during the earlier time point (T1). This observed change in NifW:NifD/K ratio could be a strategy whereby NifW acts to maintain nitrogenase functionality under conditions of high oxidative stress during the middle of the photoperiod and, for *Trichodesmium,* the purported active N_2_-fixation period [12]. These conditions are typical of diazotrophs undergoing Fe stress [13] where such a strategy may help reduce nitrogenase’s absolute Fe cost.

RuBisCO

As with the nitrogenase stoichiometry, the ratio of the RuBisCO subunits in Fe-replete conditions is near to the expected stoichiometry of 8 RbcS : 8 RbcL (S3 Fig.). Fe-deplete samples however show a distinct departure from this idealised stoichiometry and instead are observed at 10:5 stoichiometry of RbcS:RbcL. Neither subunit contains any Fe so, if functionally significant, the reason for this change in stoichiometry is of interest.

**Supplementary References**

1. Bricker TM, Roose JL, Fagerlund RD, Frankel LK, Eaton-Rye JJ (2012) The extrinsic proteins of Photosystem II. Biochim Biophys Acta 1817: 121–142. doi:10.1016/j.bbabio.2011.07.006.

2. Popelkova H, Yocum CF (2011) PsbO, the manganese-stabilizing protein: Analysis of the structure–function relations that provide insights into its role in photosystem II. Journal of Photochemistry and Photobiology B: Biology 104: 179–190. doi:10.1016/j.jphotobiol.2011.01.015.

3. Murray JW (2011) Sequence variation at the oxygen-evolving centre of photosystem II: a new class of “rogue” cyanobacterial D1 proteins. Photosynth Res 110: 177–184. doi:10.1007/s11120-011-9714-5.

4. Wegener KM, Nagarajan A, Pakrasi HB (2014) An Atypical psbAGene Encodes a Sentinel D1 Protein to Form a Physiologically Relevant Inactive Photosystem II Complex in Cyanobacteria. Journal of Biological Chemistry: jbc.M114.604124. doi:10.1074/jbc.M114.604124.

5. Pfreundt U, Kopf M, Belkin N, Berman-Frank I, Hess WR (2014) The primary transcriptome of the marine diazotroph Trichodesmium erythraeum IMS101. Sci Rep 4: 6187. doi:10.1038/srep06187.

6. Baniulis D, Yamashita E, Whitelegge JP, Zatsman AI, Hendrich MP, et al. (2009) Structure-Function, Stability, and Chemical Modification of the Cyanobacterial Cytochrome b6f Complex from Nostoc sp. PCC 7120. Journal of Biological Chemistry 284: 9861–9869. doi:10.1074/jbc.M809196200.

7. Golbeck J (1992) Structure and Function of Photosystem I. Annu Rev Plant Physiol Plant Mol Biol 43: 293–324. doi:10.1146/annurev.arplant.43.1.293.

8. Xia Z, Broadhurst RW, Laue ED, Bryant DA, Golbeck JH, et al. (1998) Structure and properties in solution of PsaD, an extrinsic polypeptide of photosystem I. Eur J Biochem 255: 309–316.

9. Jordan P, Fromme P, Witt HT, Klukas O, Saenger W, et al. (2001) Three-dimensional structure of cyanobacterial photosystem I at 2.5 angstrom resolution. Nature 411: 909–917. doi:10.1038/35082000.

10. Kustka A, Sanudo-Wilhelmy S, Carpenter EJ, Capone DG, Raven JA (2003) A revised estimate of the iron use efficiency of nitrogen fixation, with special reference to the marine cyanobacterium Trichodesmium spp. (Cyanophyta). J Phycol 39: 12–25. doi:10.1046/j.1529-8817.2003.01156.x.

11. Kim S, Burgess BK (1996) Evidence for the direct interaction of the nifW gene product with the MoFe protein. Journal of Biological Chemistry 271: 9764–9770.

12. Berman-Frank I, Lundgren P, Chen YB, Küpper H, Kolber Z, et al. (2001) Segregation of nitrogen fixation and oxygenic photosynthesis in the marine cyanobacterium Trichodesmium. Science 294: 1534–1537. doi:10.1126/science.1064082.

13. Latifi A, Jeanjean R, Lemeille S, Havaux M, Zhang C-C (2005) Iron starvation leads to oxidative stress in Anabaena sp. strain PCC 7120. Journal of Bacteriology 187: 6596–6598. doi:10.1128/JB.187.18.6596-6598.2005.
